# Neural oscillations and connectivity characterizing the state of tonic experimental pain in humans

**DOI:** 10.1002/hbm.24784

**Published:** 2019-09-09

**Authors:** Moritz M. Nickel, Son Ta Dinh, Elisabeth S. May, Laura Tiemann, Vanessa D. Hohn, Joachim Gross, Markus Ploner

**Affiliations:** ^1^ Department of Neurology and TUM‐Neuroimaging Center TUM School of Medicine, Technical University of Munich Munich Germany; ^2^ Institute of Neuroscience and Psychology University of Glasgow Glasgow UK; ^3^ Institute for Biomagnetism and Biosignalanalysis University of Münster Münster Germany

**Keywords:** brain networks, electroencephalography, functional connectivity, graph theory, neuronal oscillations, tonic pain

## Abstract

Pain is a complex phenomenon that is served by neural oscillations and connectivity involving different brain areas and frequencies. Here, we aimed to systematically and comprehensively assess the pattern of neural oscillations and connectivity characterizing the state of tonic experimental pain in humans. To this end, we applied 10‐min heat pain stimuli consecutively to the right and left hand of 39 healthy participants and recorded electroencephalography. We systematically analyzed global and local measures of oscillatory brain activity, connectivity, and graph theory‐based network measures during tonic pain and compared them to a nonpainful control condition. Local measures showed suppressions of oscillatory activity at alpha frequencies together with stronger connectivity at alpha and beta frequencies in sensorimotor areas during tonic pain. Furthermore, sensorimotor areas contralateral to stimulation showed significantly increased connectivity to a common area in the medial prefrontal cortex at alpha frequencies. Together, these observations indicate that the state of tonic experimental pain is associated with a sensorimotor‐prefrontal network connected at alpha frequencies. These findings represent a step further toward understanding the brain mechanisms underlying long‐lasting pain states in health and disease.

## 1 INTRODUCTION

Pain is a complex phenomenon which serves to protect the body. In contrast, ongoing pain in chronic pain syndromes does no longer serve protective functions but represents a pathological condition with detrimental effects on quality of life. In the brain, the experience of pain is associated with the activation of an extended network of brain areas (Baliki & Apkarian, 2015; Garcia‐Larrea & Peyron, 2013). This network yields pain‐related neural oscillations at different frequencies ranging from infra‐slow oscillations below 0.1 Hz to theta (4–8 Hz), alpha (8–13 Hz), beta (13–29 Hz), and gamma (30–100 Hz) frequencies (Ploner, Sorg, & Gross, 2017). In addition, pain depends on the communication between different brain areas, that is, on functional connectivity (Apkarian & Chialvo, 2006; Kucyi & Davis, 2015; Mano & Seymour, 2015; Tracey, 2005). Functional connectivity can be observed at different frequencies, which are thought to represent complementary, for example, short‐range and long‐range or bottom‐up and top‐down, communication processes (Fries, 2015; Ploner et al., 2017; Siegel, Donner, & Engel, 2012).

So far, most evidence on the cerebral processing of pain refers to brief experimental pain stimuli whose brain mechanisms likely differ from those of tonic pain states (Baliki & Apkarian, 2015; Kuner & Flor, 2017). Comparatively few electroencephalography (EEG), magnetoencephalography (MEG), and intracranial recordings have investigated the brain processing of tonic experimental pain stimuli. They have shown local suppressions of alpha and beta oscillations in sensorimotor cortices (Colon, Liberati, & Mouraux, 2017; Giehl, Meyer‐Brandis, Kunz, & Lautenbacher, 2014; Huishi Zhang, Sohrabpour, Lu, & He, 2016; Nickel, May, Tiemann, Schmidt, et al., 2017; Nir, Sinai, Moont, Harari, & Yarnitsky, 2012; Peng, Hu, Zhang, & Hu, 2014; Schulz et al., 2015) and insula (Liberati et al., 2019) as well as increases of gamma oscillations in prefrontal areas (Nickel, May, Tiemann, Schmidt, et al., 2017; Peng et al., 2014; Schulz et al., 2015). Some studies revealed changes of the peak frequency of neural oscillations (Furman et al., 2018; Nir, Sinai, Raz, Sprecher, & Yarnitsky, 2010). Moreover, a few recent investigations have shown changes of functional connectivity at alpha and beta frequencies during tonic pain (Gram et al., 2017; Huishi Zhang et al., 2016; Levitt, Choo, Smith, LeBlanc, & Saab, 2017; Martel et al., 2017). However, these studies addressed either oscillatory brain activity or functional connectivity and either global, that is, whole‐brain summary measures, or local measures. Thus, a comprehensive picture of local and global neural oscillations and functional connectivity up to the whole‐brain network level associated with tonic experimental pain is lacking so far.

In the present study, we therefore aimed to comprehensively characterize the pattern of neural oscillations and functional connectivity reflecting the state of tonic experimental pain in humans. To this end, we applied tonic heat pain stimuli to the hands of 39 healthy participants and recorded EEG. We systematically analyzed global and local oscillatory brain activity and connectivity and graph theory‐based network measures during tonic pain and compared them to a nonpainful control condition.

## 2 METHODS

### 2.1 Participants

Fifty‐one healthy human participants (age 24.7 ± 5.6 years [mean ± *SD*], 24 female) participated in the study. All participants were right‐handed and gave written informed consent. Due to technical issues with the stimulation device, data sets of 12 participants had to be excluded, resulting in a final sample size of 39 participants (age 24.3 ± 5.6 years, 18 female). The study was approved by the ethics committee of the TUM School of Medicine of the Technical University of Munich. Other analyses of the same data set which addressed the instantaneous pain intensity rather than the state of tonic pain have been published previously (Nickel, May, Tiemann, Postorino, et al., 2017; Nickel, May, Tiemann, Schmidt, et al., 2017).

### 2.2 Paradigm

The experiment consisted of two tonic *pain* conditions and two *control* conditions. In the *pain* conditions, painful heat stimuli with duration of 10 min were applied to the dorsum of the left (*pain left*) or the right hand (*pain right*). Apart from the side of stimulation, the two *pain* conditions were identical. Left‐ and right‐hand stimulations were performed to test the replicability of findings and to disentangle stimulus location‐dependent and stimulus location‐independent neural processes. In the *control* conditions, the temperature of the stimulation device was set at 32°C, close to skin temperature. The order of the two *pain* conditions was counterbalanced across participants and *control* conditions always followed the respective *pain* condition. During all conditions, participants wore headphones playing white noise to cancel out ambient noise.

In the *pain* conditions, painful heat stimuli were applied using a thermode (TSA‐II, Medoc, Ramat Yishai, Israel). While the time course of stimulation was the same for all participants, stimulus intensity varied with a rate of 0.1°C/s between three individually adjusted temperature levels (low, medium, and high) exceeding the individual pain threshold temperature (see below) by 0.5, 0.8, or 1.1°C, respectively. The total stimulation time course consisted of 9 plateaus of 40, 50, or 60 s duration with 3 plateaus for each temperature level. During stimulation, participants continuously rated the perceived pain intensity on a visual analogue scale ranging from *no pain* to *worst tolerable pain* (0–100) using a custom‐built finger‐span device with the nonstimulated hand. Data between the start of the first temperature plateau and the end of the last decrease in stimulation temperature was included in the analysis, resulting in an 8.2 min time window for analysis. To determine the pain threshold temperature, participants were asked to adjust the stimulation temperature over the course of 3 min to their individual pain threshold before the first *pain* condition. The pain threshold was defined as the average stimulation temperature during the last 10 s of this procedure. The hand for which the threshold was assessed was counterbalanced across subjects and the same threshold was used to determine stimulus intensities for both hands. Mean pain threshold temperature was 44.7 ± 1.1°C (mean ± *SD*), resulting in an average stimulus intensity of 45.5 ± 1.1°C during the 8.2 min window of analysis. Average pain ratings of the *pain left* and *pain right* condition were 53.3 ± 23.0 and 49.6 ± 23.4, respectively. In the two *control* conditions, the thermode remained attached to the same hand as in the respective *pain* condition, but at a neutral temperature of 32°C. In these conditions, participants performed a rating procedure similar to the *pain* conditions, tracking the visually displayed and temporally inverted pain ratings of the respective *pain* conditions with the finger‐span device. For further details of the procedure please refer to Nickel, May, Tiemann, Schmidt, et al. (2017).

Stimulus presentation and timing was controlled using MATLAB (Mathworks, Natick, MA) and the Psychophysics Toolbox (http://psychtoolbox.org/).

### 2.3 Recordings and preprocessing

EEG data were recorded using an electrode montage of 64 electrodes consisting of all 10–20 system electrodes and the additional electrodes Fpz, CPz, POz, Oz, Iz, AF3/4, F5/6, FC1/2/3/4/5/6, FT7/8/9/10, C1/2/5/6, CP1/2/3/4/5/6, TP7/8/9/10, P5/6, and PO1/2/9/10, plus two electrodes below the outer canthus of each eye (Easycap, Herrsching, Germany) and BrainAmp MR plus amplifiers (Brain Products, Munich, Germany). All electrodes were referenced to FCz and grounded at AFz. The EEG was sampled at 1000 Hz (0.1 μV resolution) and band‐pass filtered between 0.016 and 250 Hz. Impedances were kept below 20 kΩ.

Preprocessing was performed using BrainVision Analyzer software (Brain Products). EEG data were downsampled to 500 Hz. To detect artifacts and to compute independent component (IC) weights, a 1 Hz high‐pass filter and a 50 Hz notch filter removing line noise were applied. IC analysis was applied to the filtered EEG data after concatenation of all conditions and ICs representing eye movements and muscle artifacts were identified (Jung et al., 2000). Data segments of 400 ms around data points with amplitudes exceeding ±100 μV and data jumps exceeding 50 μV were marked for rejection. Subsequently, the identified ICs were subtracted from the unfiltered EEG and all electrodes were re‐referenced to the average reference. Remaining artifacts were identified by visual inspection and manually marked for rejection. No significant differences in relative portions of rejected data were found between conditions (*pain left/right*, 5.7 ± 3.9%, 5.2 ± 4.8%, *control left/right*, 5.0 ± 3.6%, 5.9 ± 5.1%, one‐way repeated measures ANOVA, *F*_[2, 86]_ = 0.95, *p* > .4, Greenhouse–Geisser corrected).

All further analysis steps were performed in MATLAB using the FieldTrip toolbox (Oostenveld, Fries, Maris, & Schoffelen, 2011), the Brain connectivity toolbox (Rubinov & Sporns, 2010), and custom‐written scripts. In FieldTrip, previously marked artifacts were rejected and data were downsampled to 250 Hz to accelerate further analysis. The continuous EEG data were segmented into 2 s epochs with 50% overlap which served as basis for all following analyses except for the peak frequency analysis, in which case 5 s epochs with 50% overlap were used to increase frequency resolution. All data were analyzed separately for each condition. Frequency bands were defined as follows: theta, 4–8 Hz; alpha, 8–13 Hz; beta, 14–30 Hz; gamma, 60–100 Hz. For the gamma band, frequencies between 60 and 100 Hz were chosen as previous studies (Nickel, May, Tiemann, Schmidt, et al., 2017; Peng et al., 2014; Schulz et al., 2015) showed strongest effects related to tonic pain at these frequencies.

### 2.4 Analysis—Overview

**Figure 1:**
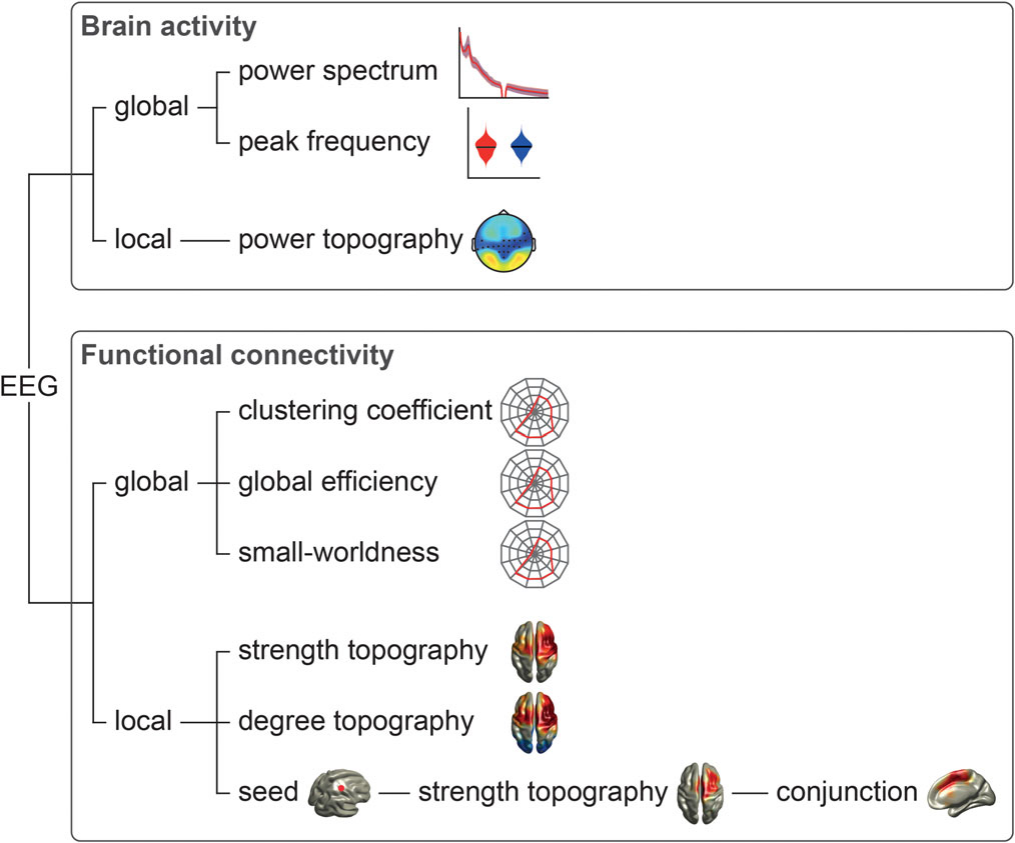
Analysis outline. EEG data were analyzed with respect to oscillatory brain activity and functional connectivity, as measured by phase locking value (Lachaux, Rodriguez, Martinerie, & Varela, 1999) and debiased weighted phase lag index (Vinck, Oostenveld, van Wingerden, Battaglia, & Pennartz, 2011). For both phenomena, global and local measures were computed. EEG, electroencephalography

Figure 1 gives an overview of the analysis, which comprised assessments of oscillatory brain activity and functional connectivity. For both, we define two categories of analyses, *global* and *local* analyses. Global analyses include analyses which average or summarize across electrodes or voxels, such as power spectra, peak frequency, and all global graph measures. Hence, global analyses are spatially unspecific. In contrast, local analyses comprise the comparison of topographies of power, strength of connectivity, and degree as local graph measure on electrode and source level. Thus, these analyses are spatially specific.

### 2.5 Analysis of oscillatory brain activity

All analyses of oscillatory brain activity were performed on electrode level. We first assessed global measures of oscillatory brain activity at frequencies between 1 and 100 Hz. To calculate global power spectra, a power spectrum with a frequency resolution of 0.5 Hz was computed using a fast Fourier transformation and Slepian multitapers with ±1 Hz frequency smoothing for each epoch and electrode. To suppress line noise, a band‐stop filter of 48–52 Hz was applied. Subsequently, the power spectra were averaged across epochs and electrodes, resulting in a global power spectrum for each condition and participant. Furthermore, we computed relative power spectra by dividing the absolute power spectra by the summed power across the whole frequency range (1–100 Hz) for each participant in order to enhance sensitivity with respect to frequency band‐specific changes in oscillatory brain activity. To determine global peak frequencies of oscillatory brain activity, a frequency analysis similar to the global absolute power spectra but with 0.2 Hz frequency resolution was performed in an extended alpha band range (6–14 Hz) (Bazanova & Vernon, 2014). Peak frequencies were determined for each epoch by detecting the frequency between 6 and 14 Hz which showed a maximum in power and subsequently averaged across epochs for each condition and participant to avoid a bias toward epochs with high alpha power (Furman et al., 2018).

To assess local measures of oscillatory brain activity, we calculated topographies of absolute and relative power for theta, alpha, beta, and gamma frequency bands for each condition and participant. Topographies of relative power were computed by dividing the absolute power spectra by the summed power across the whole frequency range (1–100 Hz) for each electrode and participant. Relative power spectra were averaged across the respective frequency ranges to obtain the relative power of each frequency band.

### 2.6 Analysis of functional connectivity

Functional connectivity was assessed using the phase locking value (PLV) (Lachaux et al., 1999), which is defined as

$$\mathrm{PLV}=\frac{1}{N}\left|\sum_{j=1}^{N}\exp^{i\left(\Phi_{x}\left(j\right)-\Phi_{y}\left(j\right)\right)}\right|.$$

Φ_*x*_(*j*) − Φ_*y*_(*j*) indicates the phase difference between two sites *x* and *y* of epoch *j*. *N* signifies the number of epochs across time. The PLV is well‐established, highly sensitive and captures zero‐phase lag and nonzero‐phase lag connectivity. In addition, we calculated the debiased weighted phase lag index (dwPLI) (Vinck et al., 2011), which is defined between two sites *x* and *y* as

dwPLI=∑j=1N∑k≠jImSxyjImSxyk∑j=1N∑k≠jImSxyjImSxyk.

*Im*{*S*_*xyj*_} and *Im*{*S*_*xyk*_} indicate the imaginary part of the cross‐spectral density *S* of epochs *j* and *k*, respectively. *N* signifies the number of 2 s epochs across time. The dwPLI captures nonzero‐phase lag connectivity only. Therefore, the dwPLI is not susceptible to volume conduction but has a reduced sensitivity, since real synchrony at zero‐phase lag is also discarded.

All connectivity analyses were performed in source space as contrasts between tonic *pain* and respective *control* conditions which reduces possible confounds by volume conduction and field spread (Schoffelen & Gross, 2009). We used linearly constrained minimum variance beamforming (Van Veen, van Drongelen, Yuchtman, & Suzuki, 1997) to project band‐pass filtered EEG data from electrode into source space. EEG data were band‐pass filtered in the predefined frequency bands using a fourth‐order Butterworth filter (forward and backward). Spatial filters were computed based on the covariance matrices of the band‐pass filtered data for each frequency band and a lead field matrix. A three‐dimensional grid with a 1 cm resolution covering the brain was defined. The lead field was constructed for each voxel using a realistically shaped three‐shell boundary‐element volume conduction model based on the template Montreal Neurological Institute (MNI) brain. We used a regularization parameter of 5% of the covariance matrix and chose the dipole orientation of most variance using singular value decomposition. Finally, the preprocessed, band‐pass filtered EEG data were projected through the spatial filter to extract the time series of neuronal activity of each frequency band at each voxel.

For global connectivity analyses, the connectivity between every pair of voxels was computed resulting in a 2020 × 2020 voxels connectivity matrix representing the strength of connection for each pair of voxels over the complete 8.2 min analysis window. To describe brain networks, we applied a graph theory‐based approach. Graph theory is a valuable framework to better understand the characteristics of complex systems, including brain networks, as it allows to describe and quantify key features of such systems in a few measures. A graph is a mathematical construct consisting of nodes and edges connecting the nodes to each other. Here, nodes were defined as voxels and edges as connectivity between voxels. To reduce the computational load, the adjacency matrix, which defines all edges between the nodes, was thresholded to the 10% strongest connections and binarized (Mano et al., 2018; Mansour et al., 2016), resulting in an unweighted and undirected graph.

In principle, graph measures can characterize either complete networks or single nodes of the network. Three complementary measures of the complete network were included in the analysis: the global clustering coefficient, global efficiency, and small‐worldness. The global clustering coefficient is the average of the local clustering coefficients of all nodes, that is, the fraction of the node's neighbors which are also connected to each other (Watts & Strogatz, 1998). It captures the prevalence of clustered connections around single nodes in a network. This is commonly interpreted as a measure of functional segregation, indicating specialized subregions of the brain which are densely connected (Rubinov & Sporns, 2010). Global efficiency is the inverse of the average shortest path length, that is, the minimum number of edges necessary to connect any pair of nodes in a network. It reflects long‐range connections which play a pivotal role for the global integration of a network (Stam, 2014). Small‐worldness describes the ratio of clustering coefficient and global efficiency in comparison to random networks (Rubinov & Sporns, 2010). Brain networks characteristically show small‐world properties, that is, clustered local connectivity with relatively few long‐range connections which renders the network efficient and minimizes wiring costs (Achard & Bullmore, 2007; Bullmore & Sporns, 2012).

To assess local connectivity, we first determined the average connectivity of each voxel to all other voxels, that is, the strength of connectivity. Second, we investigated the graph measure degree. The degree quantifies the number of nodes connected to an individual node by edges and thus, reflects the importance and the connection strength of a node in a network (Rubinov & Sporns, 2010). In the present study, edges were defined as the 10% strongest connections. Third, we analyzed to which brain regions selected brain areas, which show increased connectivity during tonic pain, are connected to in a seed‐based approach. As we found increased connectivity and degree in the contralateral sensorimotor area in both *pain* conditions and in light of previous findings (Nickel, May, Tiemann, Schmidt, et al., 2017; Schulz et al., 2015), we defined one voxel located in the primary somatosensory cortex (S1) contralateral to the stimulation (MNI_*xyz*_ = [30/−30, −30, 60] mm [Bradley, Bastuji, & Garcia‐Larrea, 2017]) as seed voxel and computed the seed‐based strength of connectivity. Moreover, we computed the individual mean and group mean phase differences in the alpha band between oscillatory activity in contralateral S1‐voxels and a region‐of‐interest in the medial frontal cortex (MNI_*xyz*_ = [0, −10, 60]) obtained as the result of the conjunction analysis with maximal *t* value (see below). Finally, to establish the dominant directionality of the seed‐based connectivity, we calculated partial directed coherence (PDC) (Baccala & Sameshima, 2001) in the alpha band as a measure of directed connectivity between the aforementioned seed voxels in the contralateral S1 and the region‐of‐interest in the medial frontal cortex.

### 2.7 Statistical analysis

Statistical analysis was carried out using FieldTrip (Oostenveld et al., 2011) and custom‐written MATLAB scripts. We used cluster‐based permutation tests with 10,000 permutations to compare each *pain* condition to the respective *control* condition (Maris & Oostenveld, 2007), either clustering across frequencies for the power spectra as part of the global analysis or clustering across space (electrodes or voxels) for all local analyses. We contrasted *pain* and *control* conditions using dependent‐samples *t* tests for every frequency/electrode/voxel and applied a cluster‐threshold at *p* < .05. Neighboring *t* values which exceeded this *p* value were summed up. Subsequently, single‐subject data were randomly assigned to the *pain* and *control* conditions and the aforementioned procedure was repeated. For each of the 10,000 permutations, the maximum summed *t* value was kept building the distribution under the null hypothesis that *pain* and *control* conditions did not differ. Statistical significance was tested with an alpha level of .05 for one‐ and two‐tailed testing. One‐tailed testing was used for local power and the seed‐based analyses only, as directed hypotheses existed, such as a decrease in contralateral alpha activity when heat pain was applied. Except for the power spectra, all global measures were compared using a nonparametric permutation test permuting single‐subject values 10,000 times between *pain* conditions and the respective *control* conditions, computing a dependent‐samples *t* test and obtaining a null distribution of *t* values to which the *t* value of the original data was compared. The alpha level was also set to .05 (two‐tailed). Bonferroni corrections were performed across the four frequency bands. With regard to the seed‐based analysis, we additionally performed a conjunction analysis (Nichols, Brett, Andersson, Wager, & Poline, 2005) of left and right‐hand stimulations to assess brain areas whose connectivity are consistently changed during tonic pain regardless of stimulation side. Phase differences between voxels in the contralateral S1 (MNI_*xyz*_ = [30/−30, −30, 60]) and medial frontal cortex (MNI_*xyz*_ = [0, −10, 60]) were statistically compared against zero using the circ_mtest.m function of the CircStat toolbox for circular statistics (Berens, 2009). Moreover, we compared PLV‐based connectivity between these voxels while controlling for alpha power at central electrodes C1/C3 and C2/C4 contralateral to stimulation to test whether the connectivity effect can be explained by alpha power. We performed repeated measures analyses of covariance (rmANCOVAs) including the factor condition (*pain* vs. *control* condition), the covariate alpha power and the dependent variable connectivity. Separate rmANCOVAs were calculated for the *pain left* and *pain right* contrasts. For this analysis, connectivity and power values were *z*‐transformed across *pain left* and *control left* conditions and across *pain right* and *control right* conditions. Finally, to test the dominant directionality of seed‐based connectivity, we contrasted the PDC between both directions (S1 to medial frontal cortex and medial frontal cortex to S1) for each *pain* condition separately using a nonparametric permutation test permuting single‐subject values 10,000 times between both connectivity directions.

## 3 RESULTS

### 3.1 Oscillatory brain activity during the tonic pain state

**Figure 2:**
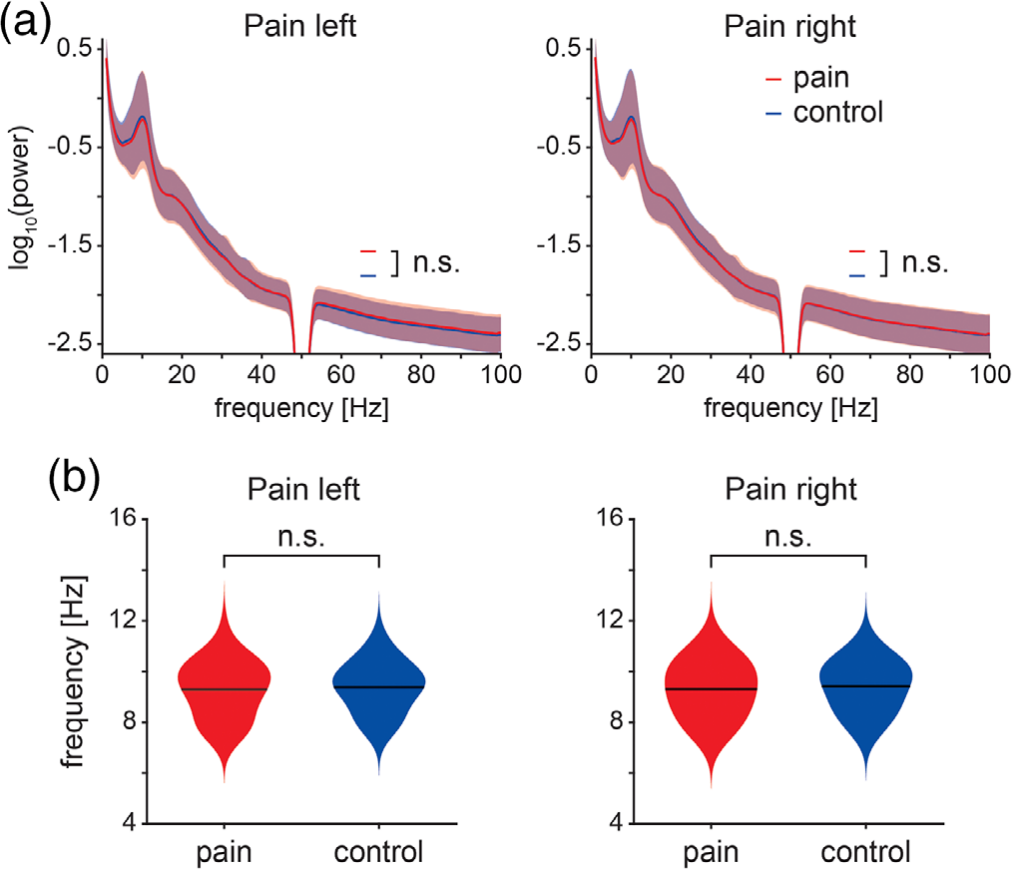
Global measures of oscillatory brain activity. (a) Absolute power spectra of *pain* (red) and *control* conditions (blue) for stimulation of the left and right hand. Individual power spectra were log‐transformed and averaged for visualization. The red and the blue shading indicate the *SD* across subjects. Cluster‐based permutation tests showed no significant differences between *pain* and respective *control* conditions. (b) Violin plots of the dominant peak frequencies of *pain* (red) and *control* (blue) conditions. Peak frequencies between 6 and 14 Hz were determined on single‐trial level to avoid a bias toward trials with high alpha power. Subsequently, single‐trial peak frequencies were averaged for each participant and condition. Black lines indicate the grand average of dominant peak frequencies across participants. Nonparametric permutation tests showed no significant differences between *pain* and *control* conditions

We first assessed global changes of oscillatory brain activity during tonic pain. To this end, we compared global power spectra and peak frequencies between *pain* and *control* conditions (Figure 2). We did not observe significant differences of absolute power spectra (*p*_min_ > .08) or relative power spectra (*p*_min_ > .07) between *pain* and *control* conditions. Similarly, peak frequencies did not differ between *pain left* and *control left* (9.29 ± 1.10 Hz and 9.38 ± 0.99 Hz, respectively, *p* > .32) or between *pain right* and *control right* (9.30 ± 1.09 Hz and 9.41 ± 0.99 Hz, respectively, *p* > .14).

**Figure 3:**
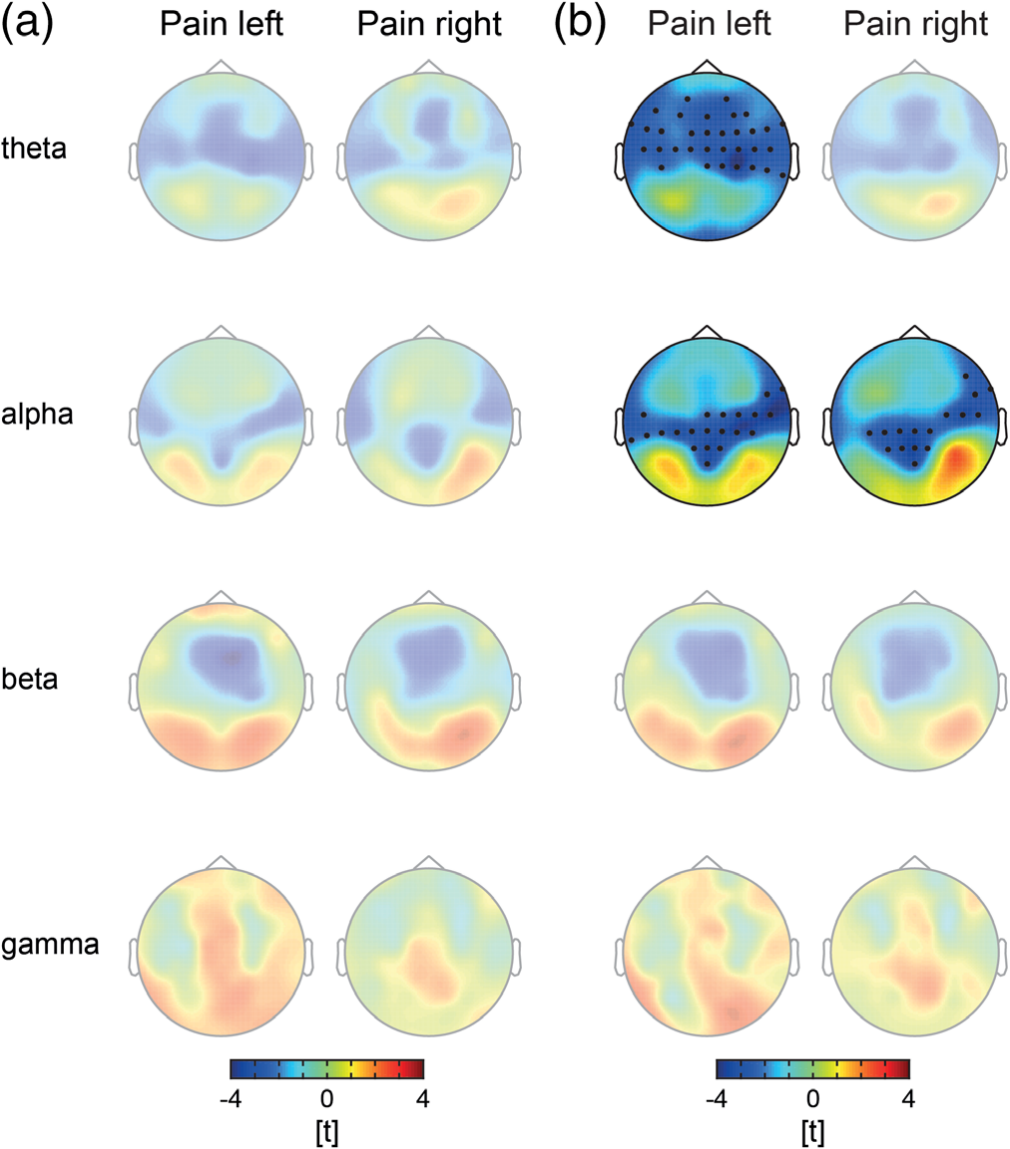
Local measures of oscillatory brain activity. (a) T‐maps of absolute power contrasts. Warm and cold colors indicate increased and decreased power in the *pain* condition as compared to the *control* condition, respectively. No significant differences in absolute power were revealed by cluster‐based permutation tests after Bonferroni correction. (b) T‐maps of relative power contrasts. Warm and cold colors indicate increased and decreased relative power in the *pain* condition as compared to the *control* condition, respectively. With regard to relative alpha power, cluster‐based permutation tests revealed significant decreases over the sensorimotor area (Bonferroni‐corrected) in both *pain* conditions. Moreover, a significant decrease in the theta frequency band was observed at fronto‐central and fronto‐lateral electrodes in the *pain left* condition. Topographies without significant cluster are presented with reduced opacity

We next analyzed local changes of oscillatory brain activity during tonic pain. We calculated topographies of absolute and relative power for *pain* and *control* conditions at theta, alpha, beta, and gamma frequency bands. Topographies of absolute power did not differ between *pain* and *control* conditions (Figure 3a; *p*_min_ > .06, Bonferroni‐corrected; see also Figure S1a). Topographies of relative power showed a significant decrease in alpha power over bilateral central electrodes covering the sensorimotor areas in *pain left* and *pain right* conditions (Figure 3b; left, *p* = .026, Cohen's *d* [*d*] = −0.31; right, *p* = .044, *d* = −0.42; Bonferroni‐corrected; see also Figure S1b). Relative theta power at frontal and central electrodes was significantly reduced in the *pain left* condition (*p* = .018, *d* = −0.40, Bonferroni‐corrected). However, we found no significant changes of relative theta power in the *pain right* condition (*p* > .21). No other significant changes in relative power were observed (*p*_min_ > .12, Bonferroni‐corrected).

### 3.2 Functional connectivity during the tonic pain state

**Figure 4:**
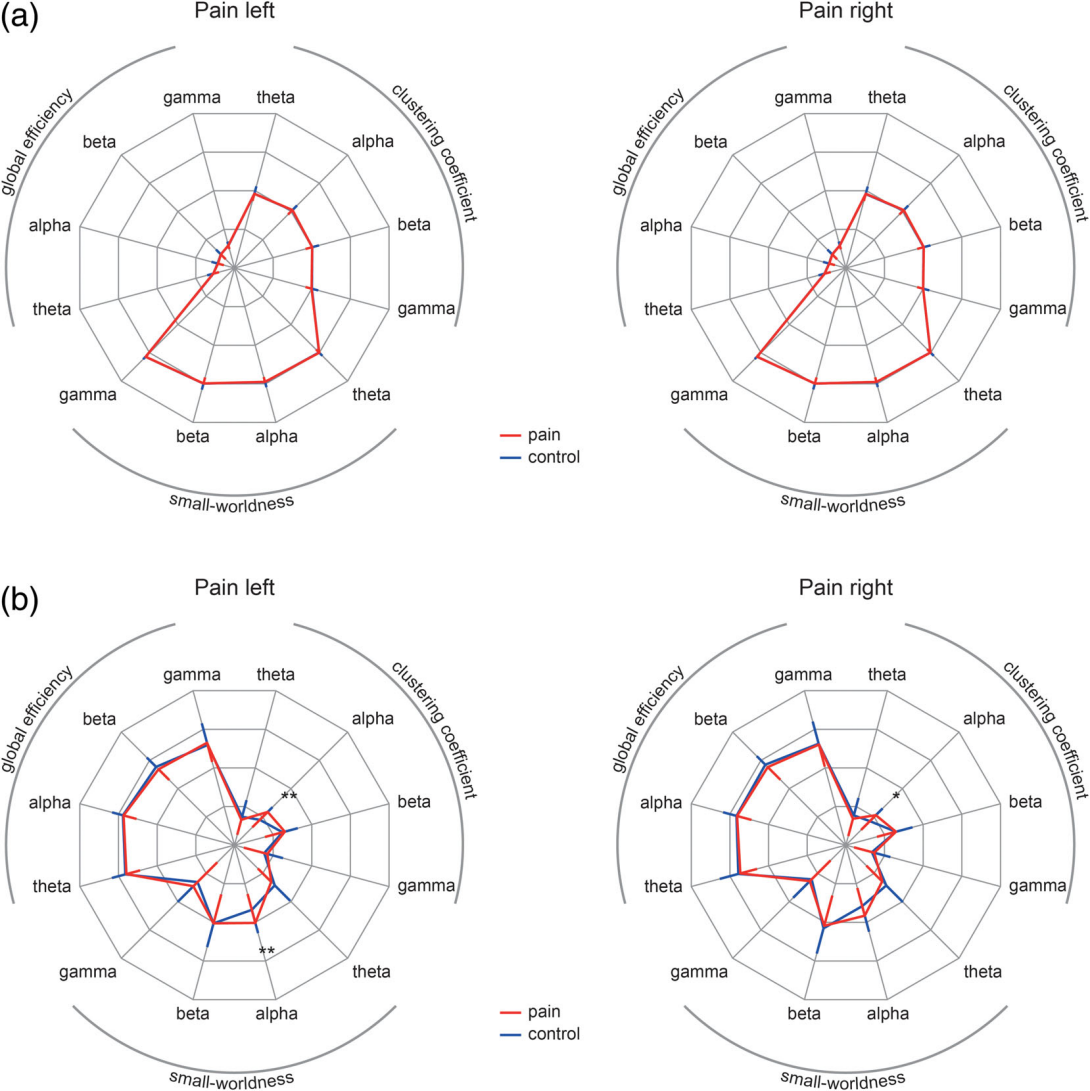
Global measures of functional connectivity. Radar charts depict global graph measures clustering coefficient, global efficiency, and small‐worldness (S) for all conditions and frequency bands. Red lines indicate *pain* conditions and blue lines indicate *control* conditions. Error bars represent the *SD* for the *pain* conditions (red, inward oriented) and *control* conditions (blue, outward oriented). (a) PLV‐based global graph measures are shown with a scale ranging from 0.4 (center point) to 0.8 in steps of 0.1. For visualization purposes, small‐worldness was scaled by a factor of 1/7. None of the PLV‐based graph measures differed significantly between *pain* and *control* conditions for any of the investigated frequency bands. (b) dwPLI‐based global graph measures are shown with a scale ranging from 0.2 (center point) to 0.6 in steps of 0.1. For visualization purposes, small‐worldness was scaled by a factor of 1/7. The global clustering coefficient in the alpha band was significantly increased in both *pain* conditions. Furthermore, small‐worldness was increased in the *pain left* condition. *p < .05, **p < .01. dwPLI, debiased weighted phase lag index; PLV, phase locking value

To investigate global changes of functional connectivity at theta, alpha, beta, and gamma frequencies during tonic pain, we performed graph theory‐based network analysis and contrasted different global graph measures between *pain* and *control* conditions. Using the PLV as connectivity measure, the global clustering coefficient (gCC), global efficiency (gEff), and small‐worldness (S) did not significantly differ between *pain* and *control* conditions in any frequency band (Figure 4a; gCC, *p*_min_ > .5; gEff, *p*_min_ > .7; S, *p*_min_ > .1, Bonferroni‐corrected). Using the dwPLI as connectivity measure (Figure 4b), the gCC in the alpha frequency band was significantly increased in both *pain* conditions (gCC, left, *p* = .0016, *d* = 0.58; right, *p* = .029, *d* = 0.45; other frequency bands, *p*_min_ > .7). Furthermore, small‐worldness in the alpha frequency was significantly enhanced in the pain left condition (S, left, *p* = .007, *d* = 0.51, Bonferroni‐corrected), but not in the pain right condition (S, right, *p* > .1, other frequency bands, *p* > .5, Bonferroni‐corrected), thus, not consistent across pain conditions. No significant differences in gEff were observed (gEff, *p*_min_ > .2, Bonferroni‐corrected).

**Figure 5:**
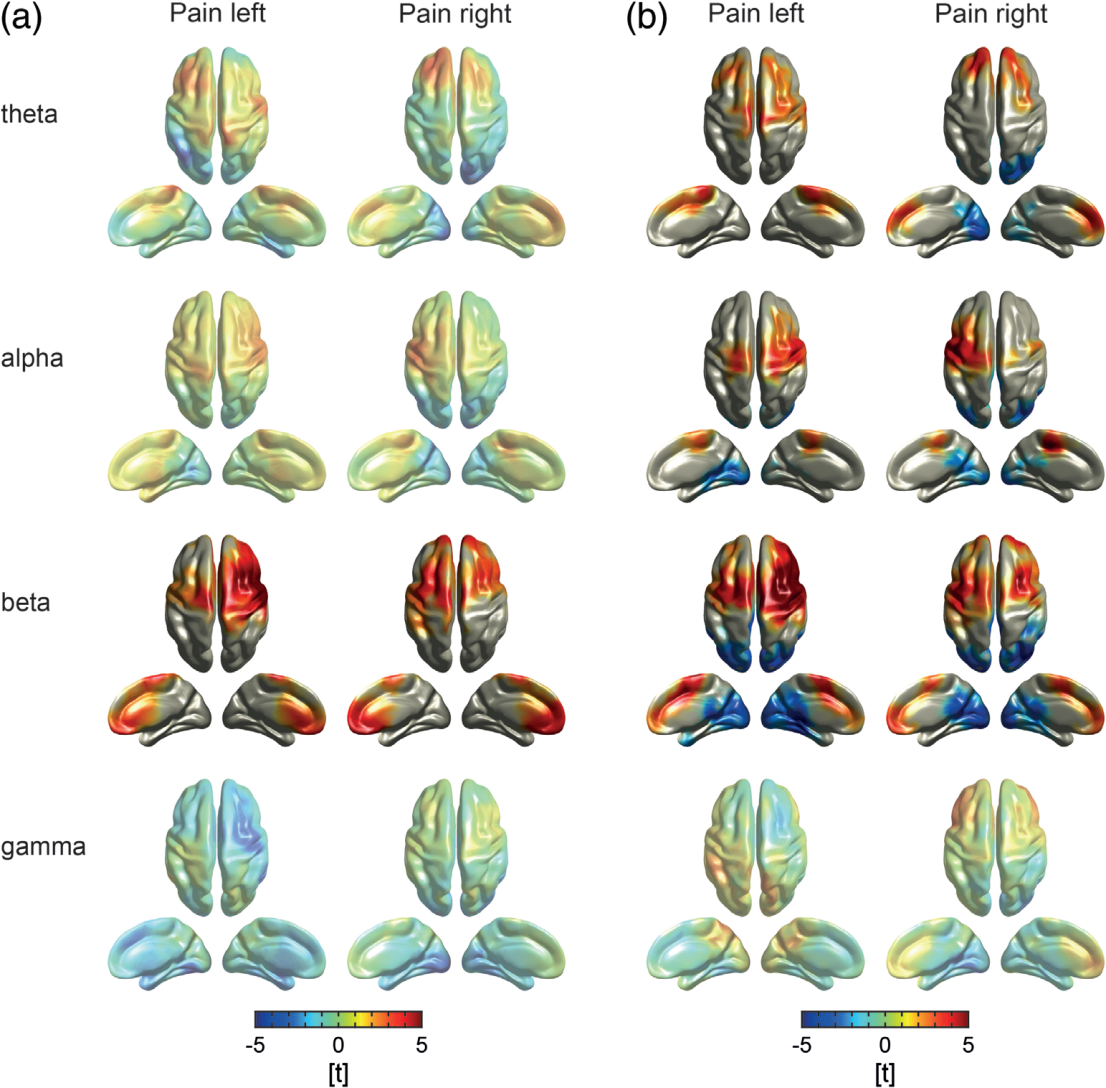
Local measures of functional connectivity. (a) T‐maps of functional connectivity contrasts between *pain* and *control* conditions. Strength of connectivity of each voxel was computed by averaging the connectivity, as measured by the PLV, of each voxel to all other voxels. Warm and cold colors indicate increased and decreased strength of connectivity in the *pain* condition as compared to the *control* condition, respectively. In the beta frequency band, connectivity was increased in both *pain* conditions predominantly contralateral to the stimulated hand (Bonferroni‐corrected). (b) T‐maps of degree contrasts between *pain* and *control* conditions. The degree of an individual node is defined by the number of nodes connected to it after thresholding to the 10% strongest connections. Warm colors indicate an enhanced degree in the *pain* condition whereas cold colors indicate lower degree in the *pain* condition as compared to the *control* condition. The contrasts revealed a higher degree in the sensorimotor cortex contralateral to the stimulation in the alpha and beta frequency bands in both *pain* conditions (Bonferroni‐corrected). In the theta frequency band, we observed an increase in degree predominantly in bilateral prefrontal cortices. Moreover, we observed a lower degree in occipital cortices at alpha and beta frequencies in both *pain* conditions (Bonferroni‐corrected). Topographies without significant cluster are presented with reduced opacity. PLV, phase locking value

We next assessed local changes of functional connectivity. We first calculated the strength of connectivity by averaging the connectivity of each voxel to all other voxels. Using the PLV, we observed an increase in connectivity in the beta frequency band predominantly contralateral to the stimulated hand in both *pain* conditions (Figure 5a; left, *p* = .0056, *d* = 0.89; right, *p* = .0056, *d* = 0.74, Bonferroni‐corrected; see also Figure S2a). No effect was observed in any other frequency band (*p*_min_ > .27, Bonferroni‐corrected). Using the dwPLI, which excludes zero‐phase lag connectivity, we did not observe any significant differences (*p*_min_ > .07, Bonferroni‐corrected; data not shown). To further investigate local changes in connectivity, we next computed the local graph measure *degree* and compared it between *pain* and *control* conditions. The degree of a node is the number of connections to other nodes after thresholding to the 10% strongest connections and therefore, conceptually related to the strength of connectivity. As shown by Figure 5b (see also Figure S2b), we found an increased degree, based on the PLV, in sensorimotor areas contralateral to the stimulation in alpha (left, *p* = .025, *d* = 0.62; right, *p* = .013, *d* = 0.98, Bonferroni‐corrected) and beta frequency bands (left, *p* < .001, *d* = 1.06; right, *p* < .001, *d* = 0.79, Bonferroni‐corrected) in both *pain* conditions. In the theta frequency band, we observed an increase in degree predominantly in the bilateral prefrontal cortices (left, *p* = .0048, *d* = 0.75; right, *p* = .026, *d* = 0.79, Bonferroni‐corrected). Moreover, significant negative occipital clusters were revealed in the alpha (right, *p* = .038, *d* = −0.65) and beta (left, *p* < .001, *d* = −0.87; right, *p* = .0016, *d* = −0.81) frequency bands, indicating fewer strong connections in the visual cortex during tonic pain as compared to the *control* condition. We found no significant differences at gamma frequencies (*p*_min_ > .41, Bonferroni‐corrected) or for the degree based on the dwPLI (*p*_min_ > .37, Bonferroni‐corrected; data not shown).

**Figure 6:**
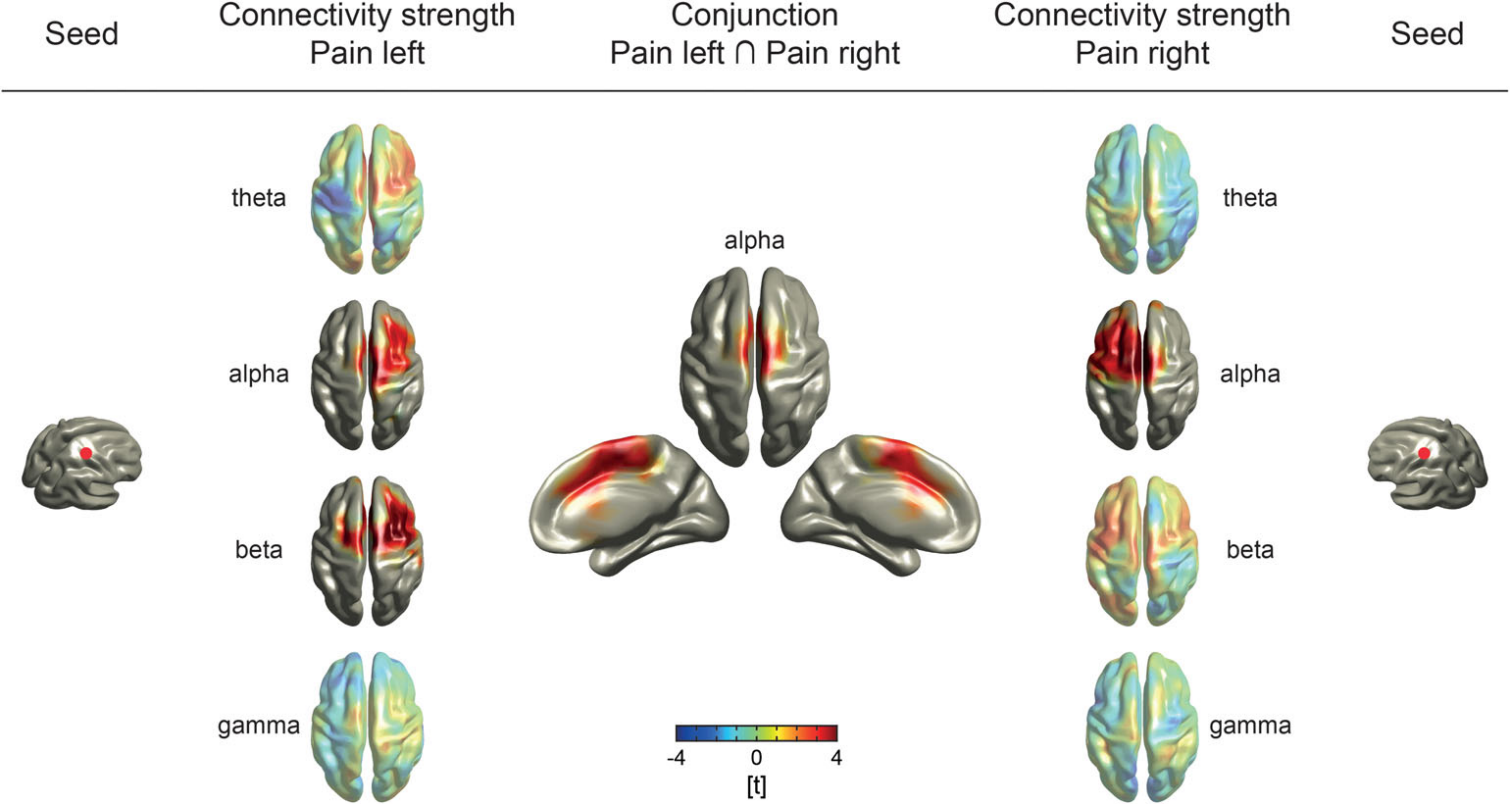
Seed‐based connectivity analysis. Functional connectivity, as measured by the PLV, was computed using voxels in the contralateral primary somatosensory cortices as seeds (MNI_*xyz*_ = [30/−30, −30, 60] [Bradley et al., 2017]). T‐maps of the seed‐based connectivity contrasts between *pain* and *control* conditions are depicted (left and right side). Warm and cold colors indicate enhanced and reduced functional connectivity in the *pain* condition as compared to the *control* condition, respectively. In both *pain* conditions, we observed increased connectivity between the somatosensory seed voxel and the medial prefrontal cortex and the frontal cortex contralateral to the stimulation side in the alpha frequency band. Moreover, a positive effect at beta frequencies was revealed in the *pain left* condition. As shown by the conjunction analysis (middle), increased alpha connectivity overlapped in the medial prefrontal cortex which indicates its involvement in the processing of tonic pain independent of the stimulation side. Topographies without significant cluster are presented with reduced opacity. PLV, phase locking value

Furthermore, we investigated to which regions brain areas with increased connectivity strength were connected during tonic pain. To this end, we used the contralateral sensorimotor area as a seed region and compared seed‐based connectivity assessed by PLV between *pain* and *control* conditions. We observed increased connectivity to the medial prefrontal cortex and the frontal cortex contralateral to the stimulation side in the alpha frequency band (Figure 6). These effects were consistent across stimulation sides (left, *p* = .030, *d* = 0.65; right, *p* < .001, *d* = 0.80, Bonferroni‐corrected). In addition, we found increased connectivity to the frontal cortex in the beta frequency band (*p* = .0044, *d* = 1.11, Bonferroni‐corrected) in the *pain left* but not in the *pain right* condition. No difference was detected at any other frequency band (*p*_min_ > .05, Bonferroni‐corrected). To determine whether the sensorimotor cortex of both hemispheres connects to a common brain area independent of stimulation side, we applied a conjunction analysis of seed‐based connectivity from both sensorimotor areas. The results revealed that both sensorimotor areas connected to the medial prefrontal cortex in the alpha frequency band during tonic pain (Figure 6, *t*_max_ = 4.7, MNI_*xyz*_ = [0, −10, 60]). The analysis of phase differences showed that they did not or only marginally differ from zero (*pain right*, 3.3 ± 15.3°, mean ± *SD*, *p* > .07; *pain left*, 4.6 ± 11.2°, *p* = .017). Using the center frequency of 10.5 Hz of the alpha band (8–13 Hz), the phase differences equaled time lags of 0.9 and 1.2 ms, respectively, indicating that connectivity was essentially observed with a phase lag close to zero.

We further investigated whether these connectivity changes between S1 and medial frontal cortex could be explained by power changes contralateral to stimulation. We therefore calculated repeated measures analysis of covariance including the factor condition, the covariate alpha power and the dependent variable connectivity. The analyses revealed significantly enhanced functional connectivity in *pain left* compared to *control left* (*F*_[1, 36]_ = 26.3, *p* < .001, partial *η* ^2^ = 0.42) and in *pain right* compared to *control right* (*F*_[1, 36]_ = 30.9, *p* < .001, partial *η* ^2^ = 0.46) independent of alpha power.

Finally, to investigate if the increased seed‐based connectivity in the alpha band assessed by PLV predominantly reflects bottom‐up or top‐down processes, we compared PDC as a measure of directed connectivity from S1 to the medial frontal cortex to the reverse direction in both *pain* conditions. We observed significantly increased PDC from S1 to the medial frontal cortex (*pain left*, *p* < .001, *d* = 0.76, *pain right*, *p* < .001, *d* = 1.28).

### 3.3 Summary

Taken together, we found a decrease of oscillatory brain activity at alpha frequencies associated with an increase of functional connectivity at alpha and beta frequencies in the contralateral sensorimotor cortex during tonic pain compared to the *control* condition. Furthermore, we found that the contralateral sensorimotor cortex showed a stronger connection to the medial prefrontal cortex during tonic pain than during the *control* condition at alpha frequencies.

## 4 DISCUSSION

In the present study, we systematically and comprehensively investigated the pattern of neural oscillations and connectivity characterizing the state of tonic experimental pain in healthy human participants. We observed local suppressions of oscillatory brain activity at alpha frequencies over the sensorimotor cortices during tonic pain. These suppressions were associated with increases of functional connectivity at alpha frequencies between sensorimotor cortices contralateral to the stimulated hand and the medial prefrontal cortex. Directed connectivity analysis suggests bottom‐up signaling of nociceptive information in the alpha frequency band from somatosensory areas to the medial prefrontal cortex. Together, these findings indicate that tonic pain is associated with local changes in oscillations and increased connectivity in a sensorimotor‐prefrontal network synchronized at alpha frequencies.

The absence of global changes of oscillatory brain activity reported here contrasts with previous studies reporting an increase (Nir et al., 2010) or a decrease (Furman et al., 2018) of the peak alpha frequency during tonic pain. Instead, our results suggest that tonic pain is more closely related to local changes of alpha oscillations over the sensorimotor cortex (see below) than to changes of global brain activity. Besides, the discrepancies might also be due to differences in control conditions. The previous studies used passive control conditions whereas we used an active control condition. As cognitive load can influence the peak alpha frequency (Haegens, Cousijn, Wallis, Harrison, & Nobre, 2014; Mierau, Klimesch, & Lefebvre, 2017), a comparison of pain to different control conditions can yield different results. In the present study, we chose an active control condition whose cognitive load was intended to match that of the pain condition.

Furthermore, we did not find global changes of functional connectivity captured by PLV‐based graph theory‐based network measures during tonic pain. This is in accordance with previous studies showing that, in healthy human participants, functional connectivity networks assessed by EEG are stable across time (Chu et al., 2012; Kramer et al., 2011). We, however, found an increased dwPLI‐based global clustering coefficient in the alpha frequency band, showing an increased functional segregation during tonic pain. Such significant changes of electrophysiological global network measures have been observed in different neurological and psychiatric disorders such as Alzheimer's disease (Stam, Jones, Nolte, Breakspear, & Scheltens, 2007; Yu et al., 2017), multiple sclerosis, and epilepsy (Stam, 2014). Although such changes have not directly been shown in chronic pain so far, fMRI studies reporting a global reorganization of the brain in chronic pain (Mano et al., 2018; Mansour et al., 2016) suggest that global changes of functional connectivity networks might also manifest in electrophysiological recordings. The present findings indicate that 10 min of experimental pain only induce changes in the dwPLI‐based global clustering coefficient. However, we only analyzed a subset of important complementary graph measures based on selected connectivity measures. Differences in other graph and connectivity measures can therefore not be ruled out.

The observation of a local suppression of alpha oscillations in sensorimotor cortices during tonic pain is in good accordance with previous studies (Colon et al., 2017; Giehl et al., 2014; Gram, Graversen, Olesen, & Drewes, 2015; Huishi Zhang et al., 2016; Nir et al., 2012; Peng et al., 2014; Schulz et al., 2015). These suppressions likely indicate activation and increased excitability (Klimesch, 2012) of the sensorimotor cortex during the processing of tonic pain. Intriguingly, our findings revealed that these local suppressions of alpha oscillations are associated with local increases of functional connectivity in the same brain areas and at the same frequencies. Compared to the small effect sizes of the changes in oscillatory brain activity, medium to large effect sizes of connectivity changes, as shown in the present study, support that connectivity plays a crucial role in the cerebral processing of pain (Apkarian & Chialvo, 2006; Kucyi & Davis, 2015; Mano & Seymour, 2015; Tracey, 2005).

The present findings further revealed that the sensorimotor cortex more strongly connected to the medial prefrontal cortex at alpha frequencies during tonic pain compared to the control condition. In light of previous findings (Nickel, May, Tiemann, Schmidt, et al., 2017), this suggests that the stimulation side‐dependent representation of stimulus intensity in sensorimotor cortex connects to a stimulation side‐independent representation of pain intensity in the medial prefrontal cortex by synchronizing at alpha frequencies. Synchrony at alpha and beta frequencies has been suggested to serve long‐range communication in the brain (Donner & Siegel, 2011). Although these frequencies have been implicated in the top‐down signaling of predictions (Fries, 2015; Michalareas et al., 2016; Ploner et al., 2017), the present findings indicate that the alpha connectivity between sensorimotor and prefrontal cortex might reflect increased bottom‐up rather than top‐down signaling of potentially nociceptive information as our recent work suggests (Nickel, May, Tiemann, Schmidt, et al., 2017). These results are in line with previous findings showing that directed functional connectivity is enhanced between somatosensory and medial frontal cortex during pain (Huishi Zhang et al., 2016). Moreover, the findings are in accordance with mounting evidence for an important role of the medial prefrontal cortex for the encoding of chronic (Baliki et al., 2006; Hashmi et al., 2013; Vachon‐Presseau et al., 2016) and tonic experimental pain (Nickel, May, Tiemann, Schmidt, et al., 2017; Schulz et al., 2015). Hub‐like properties of the medial frontal cortex with representations of pain, cognitive control, and negative emotion (Kragel et al., 2018) render it suitable for the integration of sensory and contextual information into a coherent percept and appropriate behavioral responses. The comparison of the topography of the connectivity effects in the prefrontal cortex in the present study (Figure 6) with the topography of pain‐specific fMRI activations in a recent cross‐study approach (Kragel et al., 2018) reveals an intriguing similarity of these effects. Anatomically, functional connectivity between sensorimotor and medial prefrontal cortex might be subserved by structural connections between the sensorimotor cortex and the orbital/medial prefrontal cortex as revealed by retrograde tracing studies in macaque monkeys (Carmichael & Price, 1995; Price, 2007).

Moreover, increases in connectivity were also observed in dorsolateral prefrontal cortex (DLPFC) which plays an important role in pain processing (Seminowicz & Moayedi, 2017). The DLPFC is involved in many cognitive and regulatory functions and connected to different functional brain networks (Seminowicz & Moayedi, 2017). Regarding tonic experimental pain, modulatory effects of the DLPFC on the excitability of the sensorimotor cortex were reported (De Martino, Seminowicz, Schabrun, Petrini, & Graven‐Nielsen, 2018). The present observation of increased connectivity between sensorimotor and lateral prefrontal cortex would be, in principle, well compatible with these observations of an important role of dorsolateral prefrontal‐sensorimotor connectivity in tonic pain.

Some limitations apply to the present findings and their interpretation. EEG‐based connectivity analyses using the PLV can be influenced by volume conduction and field spread effects complicating the distinction between artifactual and true connectivity effects. This particularly applies to zero‐phase lag connectivity. However, experimental (Roelfsema, Engel, Konig, & Singer, 1997; Vicente, Gollo, Mirasso, Fischer, & Pipa, 2008) and modeling (Gollo, Mirasso, Sporns, & Breakspear, 2014; Viriyopase, Bojak, Zeitler, & Gielen, 2012) evidence has indicated that physiological long‐range connectivity can occur with zero‐phase lag. Moreover, several arguments indicate that the present findings reflect physiological rather than spurious connectivity (Bastos & Schoffelen, 2016; Cohen, 2014; Palva & Palva, 2012; Schoffelen & Gross, 2009). First, we performed all connectivity analyses in source space which reduces volume conduction confounds. Second, the present results are based on contrasts between *pain* and *control* conditions which diminishes the risk of volume conduction effects (see, however, below for limitations of the *control* condition). Third, connectivity between sensorimotor and prefrontal cortex significantly differed between *pain* and *control* conditions even when controlling for power changes indicating that connectivity effects cannot be fully explained by power effects. Nevertheless, we cannot completely rule out alternative explanations for this connectivity pattern that might involve common input by one or more additional sources in combination with volume conduction effects, an inherent confound and limitation of connectivity analyses of scalp EEG recordings. Another limitation is that the *control* condition does not perfectly control for all possible confounds. In particular, *pain* and *control* conditions differ in attentional focus (body‐centered vs. object‐centered), somatosensory stimulation and salience. Hence, it is impossible to unequivocally disentangle effects of tonic pain on the one hand and attentional focus, somatosensory stimulation, and salience on the other hand (Legrain, Iannetti, Plaghki, & Mouraux, 2011). Furthermore, in the *pain* condition finger movements may have a stronger self‐initiated component than in the *control* condition in which subjects are asked to track the visually displayed pain ratings of the respective *pain* conditions. As such differences in self‐initiation can influence alpha and beta oscillations in sensorimotor cortex and the coherence between bilateral sensorimotor cortices (Wang et al., 2017), a confound by differences in self‐initiation cannot be ultimately ruled out.

## 5 CONCLUSIONS

In summary, the present findings show that tonic pain is associated with decreases of local oscillations and increases of connectivity at alpha frequencies in the sensorimotor cortex. These stimulation side‐specific changes of oscillations and connectivity occur along with increased connectivity at alpha frequencies to a stimulation side‐independent representation in the medial prefrontal cortex. Although inherent limitations in the interpretation of scalp EEG‐based connectivity analyses remain to be resolved, these findings suggest a core network for the processing of tonic pain which includes sensorimotor and medial prefrontal cortex and their synchronization at alpha frequencies. This network might be associated with the transformation of objective stimulus information into the subjective experience of pain. Together, these observations represent a step further toward understanding the cerebral representation of long‐lasting pain states and might help to identify targets for novel chronic pain treatment approaches such as noninvasive brain stimulation and neurofeedback (Jensen, Day, & Miro, 2014; Polania, Nitsche, & Ruff, 2018).

## Supporting information


**Figure S1** Local measures of oscillatory brain activity. (a) Maps of absolute power contrasts. Warm and cold colors indicate increased and decreased power in the *pain* condition as compared to the *control* condition, respectively. No significant differences in absolute power were revealed by cluster‐based permutation tests after Bonferroni correction. b. Maps of relative power contrasts. Warm and cold colors indicate increased and decreased relative power in the *pain* condition as compared to the *control* condition, respectively. With regard to relative alpha power, cluster‐based permutation tests revealed significant decreases over the sensorimotor area (Bonferroni‐corrected) in both *pain* conditions. Moreover, a significant decrease in the theta frequency band was observed at fronto‐central and fronto‐lateral electrodes in the *pain left* condition. Topographies without significant clusters are presented with reduced opacity.
**Figure S2**. Local measures of functional connectivity. a. Maps of functional connectivity contrasts between *pain* and *control* conditions. Strength of connectivity of each voxel as measured by the PLV was computed by averaging the connectivity to all other voxels. Warm and cold colors indicate increased and decreased strength of connectivity in the *pain* condition as compared to the *control* condition, respectively. In the beta frequency band, connectivity was increased in both *pain* conditions predominantly contralateral to the stimulated hand (Bonferroni‐corrected). b. Maps of degree contrasts between *pain* and *control* conditions. The degree of an individual node is defined by the number of nodes connected to it after thresholding to the 10% strongest connections. Warm colors indicate an enhanced degree in the *pain* condition whereas cold colors indicate lower degree in the *pain* condition as compared to the *control* condition. The contrasts revealed a higher degree in the sensorimotor cortex contralateral to the stimulation in the alpha and beta frequency bands in both *pain* conditions (Bonferroni‐corrected). In the theta frequency band, we observed an increase in degree predominantly in bilateral prefrontal cortices. Moreover, we observed a lower degree in occipital cortices at alpha and beta frequencies in both *pain* conditions (Bonferroni‐corrected). Topographies without significant clusters are presented with reduced opacity
