# Supplementary material for: Neural oscillations and connectivity characterizing the state of tonic experimental pain in humans
Source: Hum Brain Mapp. 2019 Sep 9;41(1):17–29. doi: 10.1002/hbm.24784 (PMC7267966; doi:10.1002/hbm.24784)
Supplement: Supplementary file 1 — Figure S1 Local measures of oscillatory brain activity. (a) Maps of absolute power contrasts. Warm and cold colors indicate increased and decreased power in the pain condition as compared to the control condition, respectively. No significant differences in absolute power were revealed by cluster‐based permutation tests after Bonferroni correction. b. Maps of relative power contrasts. Warm and cold colors indicate increased and decreased relative power in the pain condition as compared to the control condition, respectively. With regard to relative alpha power, cluster‐based permutation tests revealed significant decreases over the sensorimotor area (Bonferroni‐corrected) in both pain conditions. Moreover, a significant decrease in the theta frequency band was observed at fronto‐central and fronto‐lateral electrodes in the pain left condition. Topographies without significant clusters are presented with reduced opacity. Figure S2. Local measures of functional connectivity. a. Maps of functional connectivity contrasts between pain and control conditions. Strength of connectivity of each voxel as measured by the PLV was computed by averaging the connectivity to all other voxels. Warm and cold colors indicate increased and decreased strength of connectivity in the pain condition as compared to the control condition, respectively. In the beta frequency band, connectivity was increased in both pain conditions predominantly contralateral to the stimulated hand (Bonferroni‐corrected). b. Maps of degree contrasts between pain and control conditions. The degree of an individual node is defined by the number of nodes connected to it after thresholding to the 10% strongest connections. Warm colors indicate an enhanced degree in the pain condition whereas cold colors indicate lower degree in the pain condition as compared to the control condition. The contrasts revealed a higher degree in the sensorimotor cortex contralateral to the stimulation in the alpha and beta frequency b [file HBM-41-17-s001.pdf]

Supplementary Material for

Neural oscillations and connectivity characterizing the state of tonic experimental pain in humans

Moritz M. Nickel[1], Son Ta Dinh[1], Elisabeth S. May[1], Laura Tiemann[1], Vanessa D. Hohn[1], Joachim Gross[2,3], Markus Ploner[1]

[1]Department of Neurology and TUM-Neuroimaging Center, Technische Universität München, 81675 Munich, Germany

[2]Institute of Neuroscience and Psychology, University of Glasgow, Glasgow, G12 8QQ, United Kingdom

[3]Institute for Biomagnetism and Biosignalanalysis, University of Münster, 48149 Münster, Germany

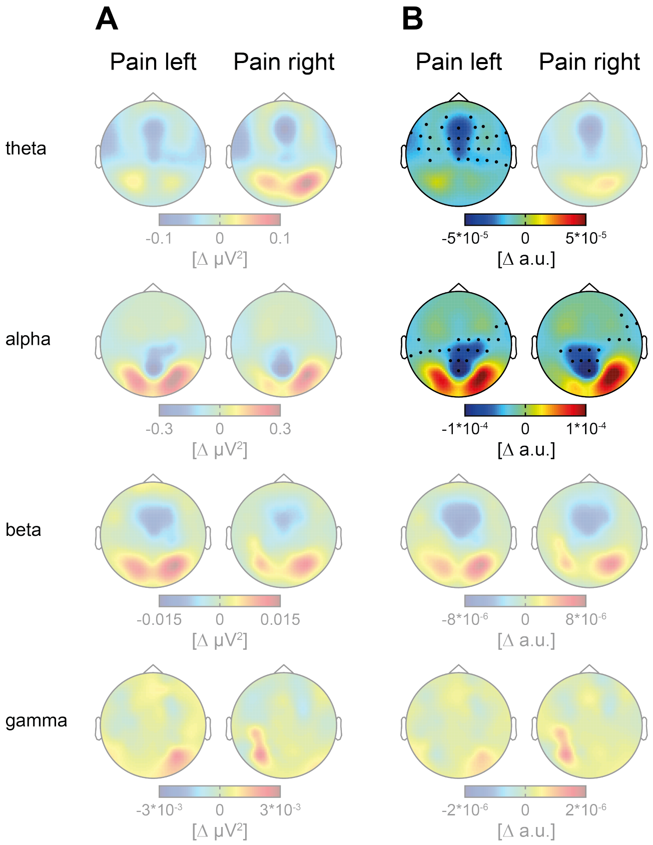

Figure S1. Local measures of oscillatory brain activity. A. Maps of absolute power contrasts. Warm and cold colors indicate increased and decreased power in the *pain* condition as compared to the *control* condition, respectively. No significant differences in absolute power were revealed by cluster-based permutation tests after Bonferroni correction. **B.** Maps of relative power contrasts. Warm and cold colors indicate increased and decreased relative power in the *pain* condition as compared to the *control* condition, respectively. With regard to relative alpha power, cluster-based permutation tests revealed significant decreases over the sensorimotor area (Bonferroni-corrected) in both *pain* conditions. Moreover, a significant decrease in the theta frequency band was observed at fronto-central and fronto-lateral electrodes in the *pain left* condition. Topographies without significant clusters are presented with reduced opacity.

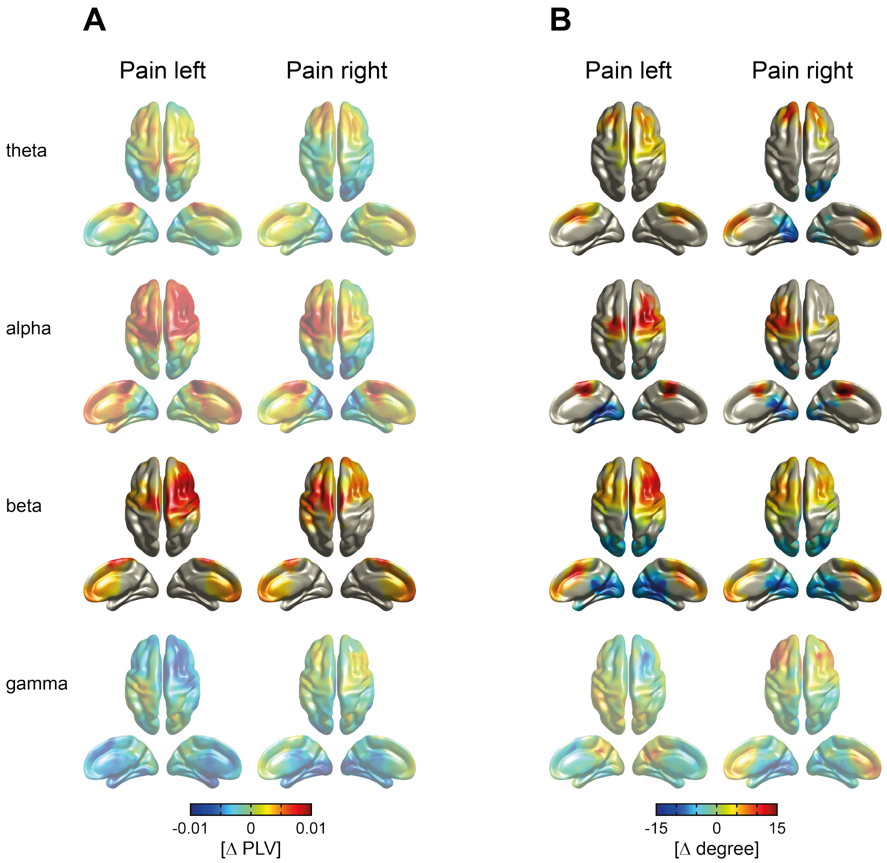

Figure S2. Local measures of functional connectivity. A. Maps of functional connectivity contrasts between *pain* and *control* conditions. Strength of connectivity of each voxel as measured by the PLV was computed by averaging the connectivity to all other voxels. Warm and cold colors indicate increased and decreased strength of connectivity in the *pain* condition as compared to the *control* condition, respectively. In the beta frequency band, connectivity was increased in both *pain* conditions predominantly contralateral to the stimulated hand (Bonferroni-corrected). **B.** Maps of degree contrasts between *pain* and *control* conditions. The degree of an individual node is defined by the number of nodes connected to it after thresholding to the 10% strongest connections. Warm colors indicate an enhanced degree in the *pain* condition whereas cold colors indicate lower degree in the *pain* condition as compared to the *control* condition. The contrasts revealed a higher degree in the sensorimotor cortex contralateral to the stimulation in the alpha and beta frequency bands in both *pain* conditions (Bonferroni-corrected). In the theta frequency band, we observed an increase in degree predominantly in bilateral prefrontal cortices. Moreover, we observed a lower degree in occipital cortices at alpha and beta frequencies in both *pain* conditions (Bonferroni-corrected). Topographies without significant clusters are presented with reduced opacity.
